# Xrp1 is a transcription factor required for cell competition-driven elimination of loser cells

**DOI:** 10.1038/s41598-018-36277-4

**Published:** 2018-12-07

**Authors:** Ludovic Baillon, Federico Germani, Claudia Rockel, Jochen Hilchenbach, Konrad Basler

**Affiliations:** Institute of Molecular Life Sciences, University of Zurich, Zurich, Switzerland

## Abstract

The elimination of unfit cells from a tissue is a process known in *Drosophila* and mammals as cell competition. In a well-studied paradigm “loser” cells that are heterozygous mutant for a haploinsufficient ribosomal protein gene are eliminated from developing tissues via apoptosis when surrounded by fitter wild-type cells, referred to as “winner” cells. However, the mechanisms underlying the induction of this phenomenon are not fully understood. Here we report that a CCAAT-Enhancer-Binding Protein (C/EBP), Xrp1, which is known to help maintaining genomic stability after genotoxic stress, is necessary for the elimination of loser clones in cell competition. In loser cells, *Xrp1* is transcriptionally upregulated by an autoregulatory loop and is able to trigger apoptosis - driving cell elimination. We further show that Xrp1 acts in the nucleus to regulate the transcription of several genes that have been previously involved in cell competition. We therefore speculate that Xrp1 might play a fundamental role as a molecular caretaker of the genomic integrity of tissues.

## Introduction

Tissues are composed by genetically heterogeneous cells as a result of the accumulation of different mutations over time. Unfit and potentially detrimental cells are eliminated from tissues via apoptosis triggered by a process known in both insects and mammals as cell competition^1,2^. The eliminated cells, referred to as “loser” cells, are normally viable and capable of growing, but are eliminated when surrounded by fitter, “winner” cells. In *Drosophila melanogaster*, the majority of ribosomal protein genes (RPGs) are haploinsufficient (hRPGs). When one copy of an hRPG is removed, this gives rise to the “Minute” phenotype characterized by a general developmental delay and improper bristle development^3^. When intermingled with wild-type winner cells, cells heterozygous for an hRPG become losers and are eliminated via apoptosis^4^. Various genetic manipulations of a tissue can result in different and widely documented cell competition responses. Several pathways, such as the BMP^5,6^, Toll^7^, Wnt^8,9^, JAK/STAT^10^, Ras/MAPK^11,12^ and Hippo pathways^13,14^, have been implicated in cell competition, suggesting the existence of a complex framework of actions that serve to induce apoptosis and eliminate loser cells. However, what actually triggers elimination yet remains elusive.

Multicellular organisms maintain genomic stability via the activation of DNA repair mechanisms to identify and correct DNA damages. During this process, cell cycle progression is arrested to prevent the expansion of damaged cells. However, when DNA repair fails, apoptosis is induced to eliminate irremediably damaged cells^15^. The p53 transcription factor plays an evolutionarily conserved role in the induction of apoptosis following DNA damage, however evidence points towards the existence of alternative routes for the induction of apoptosis in response to DNA damage^16–18^.

Here we show that, in a cell competition context, a possible alternative route to P53 for the induction of apoptosis goes via *Xrp1*, a gene encoding a b-ZIP DNA binding protein. The expression of *Xrp1* is induced in various stress conditions, for instance in response to irradiation^19–22^. Notably, *Xrp1* mutant animals have been reported to have higher levels of loss-of-heterozygosity after ionizing radiations^20^. Additionally Xrp1 plays a role in repair of DNA breaks after transposase cleavage^23^. Therefore *Xrp1* may have a role in sensing and responding to DNA damage.

Here we report the discovery, in an EMS-based screen, of *Xrp1* mutations that suppress the elimination of loser cells. This is consistent with earlier reports that proposed Xrp1 might affect cell competition^24,25^. For the first time we discern how Xrp1 might regulate cell competition. We show that Xrp1 is homologous to mammalian C/EBPs, a class of transcription factors that is known to autoregulate their own transcription^26^, to prevent proliferation and induce apoptosis. We further show that *Xrp1* expression is upregulated in loser cells in response to the removal of one copy of a haploinsufficient ribosomal protein gene, where, similarly to C/EBP homologs, it regulates its own expression via a positive autoregulatory loop, the expression of pro-apoptotic genes and that of other genes that were previously implicated in cell competition.

**Figure 1:**
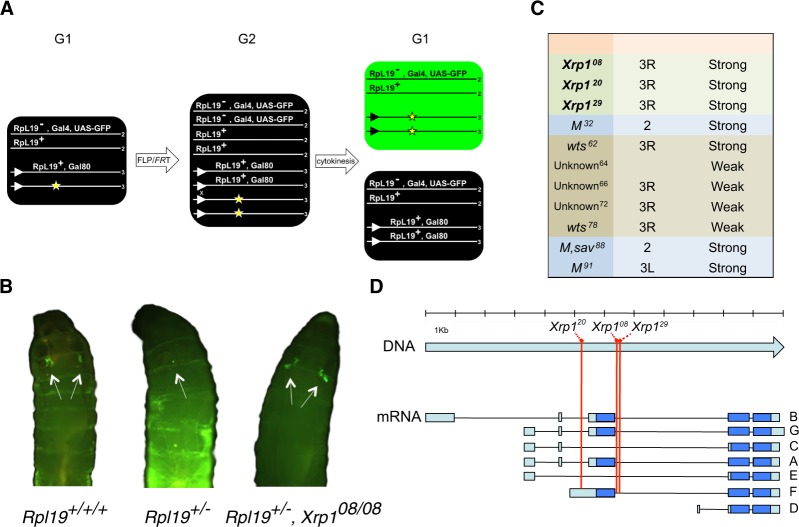
*Xrp1* mutations suppress cell competition driven elimination of loser cells in an EMS-based screen. Schematic of the genetics used to generate *RpL19*^+/−^ loser clones in a wild-type background using the FLP/FRT system. Lines represent chromosomes, numbers at the end of each line indicate the chromosome number and triangles represent FRTs on the right arm of chromosome 3. Site-directed recombination between FRTs occurs when the expression of FLP is induced via heat shock. The yellow asterisk marks the chromosome to be tested for the presence of an EMS induced suppressor. The arrangement depicted here is a variation of the classical. MARCM technology that allows us to GFP label cells that are *RpL19*^+/−^ and homozygous for a mutagenized chromosome arm 3R **(A)**. Representative examples of living larvae displaying GFP clones in the pouch of the wing imaginal discs. SalE drives Gal4 expression in the wing pouch. (Left) Positive control for clone induction using the *FRT82 RpL19*^+^ chromosome. Recombination generates *RpL19*^+/+/+^ cells that are not eliminated. (Middle) Negative control for clone induction using the isogenized FRT82 chromosome. Recombination produces *RpL19*^+/−^ cells that are efficiently eliminated. (Right) Suppressor *Xrp1*^*08*^ rescues the elimination of *RpL19*^+/−^ cells **(B)**. List of suppressive mutations retrieved with the EMS screen. Intronic mutations *Xrp1*^*08*^, *Xrp1*^20^ and *Xrp1*^29^ are strong suppressors **(C)**. Different *Xrp1* mRNA isoforms (from A to G). Blue color indicates the coding regions and light blue the untranslated regions. The red lines indicate the position of the three *Xrp1* alleles retrieved from the EMS screen (*Xrp1*^*08*^, *Xrp1*^20^ and *Xrp1*^29^) **(D)**.

In order to identify genes whose function is necessary for the elimination of *RPG* heterozygous mutant loser cells, we performed a forward genetic screen using ethyl methanesulfonate (EMS) in *Drosophila melanogaster*. We designed a mosaic system that allows direct screening through the larval cuticle for the persistence of otherwise eliminated *RpL19*^+/−^ loser clones (Fig. 1A). This enabled us to screen a high number of animals for mutations that either dominantly (anywhere in the genome) or recessively (on the right arm of the third chromosome) suppress cell competition. The induction of a single somatic recombination event between two FLP recognition targets (FRTs) generates a *RPG* heterozygous mutant cell that becomes homozygous for the mutagenized right arm of the third chromosome. Loser clones are induced at the beginning of larval development (L1). If no suppressive mutation is present, clones are efficiently eliminated over time and thus undetectable by the end of the third instar larval stage (L3) when the screening is performed (Fig. 1A). We screened 20,000 mutagenized genomes for the presence of mutations that prevent the elimination of loser clones. We retrieved 11 heritable suppressors (Fig. 1C) and focused our attention on three of the strongest suppressors that did not display any obvious growth-related phenotype. Figure 1B shows representative living larvae that were analyzed for the presence of *RpL19*^+/−^ GFP clones in the wing discs. *RpL19*^+/−^ clones are eliminated and little or no signal is observed. Their elimination, however, is prevented when cells are not heterozygous mutant for *RpL19* or when different *Xrp1* mutations (*Xrp1*^*08*^ in the example) are additionally present. In the latter cases GFP signal is observed in wing discs.

*Xrp1* suppressors did not belong to a lethal complementation group and the causative mutations were identified using a combination of positional mapping and whole-genome re-sequencing. In particular, three independent mutations in the introns of *CG17836*/*Xrp1* were identified, all caused by substitutions of single nucleotides (Fig. 1C,D). These nucleotides are conserved within the *Drosophila* genus and inspection of the alignment revealed an embedment of these nucleotides in conserved sequence motifs (Fig. S1). Of particular interest are the polypyrimidine motifs containing the nucleotide mutations in *Xrp1*^20^ and *Xrp1*^*08*^. These motifs flank the alternative first exon and are potential splice regulators. The CTCTCT motif in proximity of the 5′ splice site of Xrp1 has been identified as a putative intronic splicing enhancer (ISE) predicted to serve as binding site for the polypyrimidine-tract binding protein (PTB) splicing regulator^27^. The presence of these motifs prompted us to investigate the consequences of the *Xrp1*^*08*^ allele on exonic junctions. The most prominent effect of this allele is a strong and consistent reduction in the expression of two similar *Xrp1* transcripts, RC and RE (Fig. S1), which only differ in the composition of their 5′ UTRs. They share the transcriptional start site and contain the same long open reading frame that codes for the short isoform of Xrp1 (Fig. S1).

**Figure 2:**
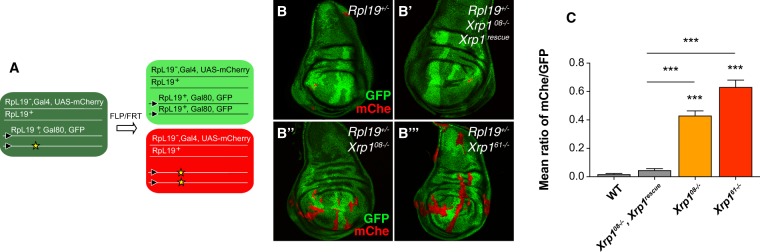
Xrp1 is required for the elimination of *RpL19*^+/−^ loser cells. Schematic representation of the twin spot MARCM system used to generate *RpL19*^+/−^ loser clones (red) and their respective twin spot clones (bright green, two copies of GFP) in a background of wild-type GFP positive cells (dark green) **(A)**. *RpL19*^+/−^ loser cells are eliminated (B). *RpL19*^+/−^ cells are eliminated when *Xrp1* mutations are rescued with the re-introduction of one copy of *Xrp1* **(B’)**. Loser cells elimination is rescued via either intronic *Xrp1*^*08*−/−^ mutations retrieved from the EMS screen (B”) or, even more efficiently, via *Xrp1*^*61*−/−^ mutations in the coding sequence of *Xrp1* (B”’). Quantification of the mean ratio between mCherry Area and GFP^2+^ area (mChe/GFP). *Minute* loser cells, labeled with mCherry, are eliminated and the mChe/GFP ratio is close to 0. *Xrp1* mutants rescue the elimination of loser cells (ratio close to 1). ***P < 0.001, *P < 0.05, Kruskal-Wallis test. Bars represent SEM. n = 52,47,45,48. Additional significance was calculated via assessing distribution normality (D’Agostino & Pearson normality test). *Xrp1*^*08*−/−^ and *Xrp1*^*61*−/−^ follow a normal distribution (P < 0.001) **(C)**.

We then checked the behavior of *RpL19*^+/−^ clones in the presence and absence of Xrp1 function. To this end we used the twin spot MARCM system, which enables us to differently mark twin clones generated by the same recombination event. In our genetic set up, mCherry expression marks loser clones whereas two copies of GFP mark wild-type twin clones (Fig. 2A). As expected, *RpL19*^+/−^ loser clones are eliminated from the tissue (Fig. 2B). Elimination is also observed when *RpL19*^+/−^ cells within these clones are additionally mutant for *Xrp1*^*08*^ but contain a transgene comprising the genomic region of *Xrp1* (Fig. 2B’). Importantly, when *Xrp1* mutations are not rescued cell competition-driven elimination of *RpL19*^+/−^ losers no longer occurs. In particular, we show that the *Xrp1*^*08*^ intronic mutation retrieved from the EMS screen is able to prevent loser cell elimination (Fig. 2B”) and that a similar result is obtained with a newly generated complete loss-of-function allele, *Xrp1*^*61*^ (Fig. 2B”’), as well as with *Xrp1*^26^ (Fig. S4). *Xrp1*^*61*^ contains a frame shift mutation upstream of the Xrp1 basic region-leucine zipper domain (b-ZIP), and is considered a null allele. Like other *Xrp1* alleles analyzed it is homozygous viable and does not impair the development of mutant animals. To confirm that Xrp1 function is of general importance for the elimination of *hRPG*^+/−^ cells, and not limited to *RpL19*^+/−^ loser cells, we tested the effect of *Xrp1* mutations on *RpL14*^+/−^ loser clones (Fig. S2). Similarly to *RpL19*^+/−^ cells, *RpL14*^+/−^ cells are normally eliminated from wing discs during larval development. No elimination occurs if these cells express *RpL14* from a transgene, or when *Xrp1* is mutated (*Xrp1*^*61*^) (Fig. S2).

**Figure 3:**
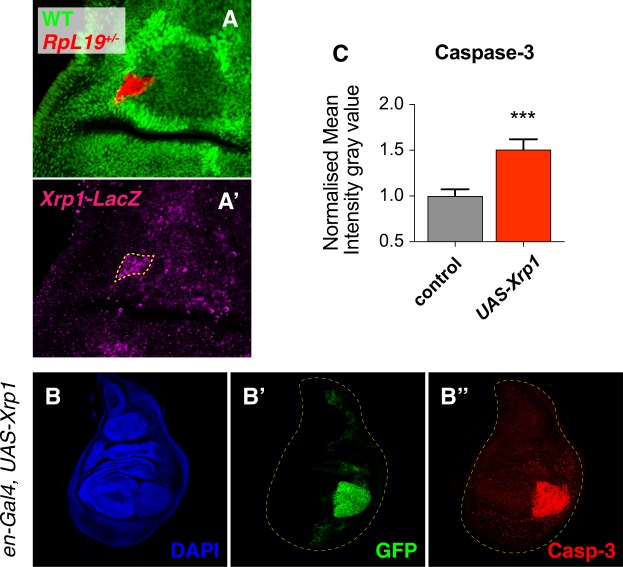
Xrp1 is upregulated in loser clones and functions as a driver of apoptosis. *Xrp1* is upregulated in *RpL19*^+/−^ loser cells as observed with a LacZ-reporter (**A**-A’). Overexpression of Xrp1 in the posterior compartment (*en-Gal4*) induces apoptosis, as observed with an anti-cleaved Caspase 3 staining (**B**-B”). Normalized quantification of the mean intensity gray value confirms increased apoptotic staining. Paired ratio t-test was applied. ***P < 0.001 (**C**).

Since *Xrp1* is transcriptionally induced in response to various forms of stress^19–22^ and since Xrp1 has been found to be upregulated in *RpS3*^+/−^ wing discs when compared to WT discs^25,28^, we hypothesized that its expression is induced in loser clones as a result of the loss of a haploinsufficient ribosomal protein gene. We therefore used a transcriptional reporter for *Xrp1* - *Xrp1*^*02515*^, containing a *lacZ* P-element^20^ - and found that *Xrp1* expression is indeed upregulated in *RpL19*^+/−^ cells, indicating that the upregulation of Xrp1 might play a crucial early role in the elimination of loser cells (Fig. 3A-A’). In line with the recent report by Lee *et al*.^25^ we found that Xrp1 is upregulated in wing discs that are lacking one copy of a ribosomal protein gene, indicating that Xrp1’s role in cell competition does not depend on clonality (Fig. S3). In order to gain insights into this function we conditionally forced the expression of Xrp1 in the posterior half of the wing discs and observed a massive induction of apoptosis, as revealed by anti-cleaved caspase 3 staining (Fig. 3B,C).

Interestingly, unlike loss of *Xrp1*, blocking apoptosis by means of overexpression of *dIAP1* or *p35*, or by abrogating the function of Dronc or Hid, does not fully suppress *RpL19*^+/−^ cell elimination, suggesting that Xrp1 does more than merely induce apoptosis. Only the co-overexpression of *CycE*, which promotes cell cycle entry, together with *dIAP1*, which suppresses apoptosis, lead to a degree of suppression of *RpL19*^+/−^ cell elimination comparable to that obtained with *Xrp1* loss-of-function mutations (Fig. S4). This indicates that the combined effects of blocking cell cycle progression and promoting apoptosis are critical for the elimination of *RPG*^+/−^ cells. Given the strength of the effect of *Xrp1* mutations, Xrp1 may therefore additionally hinder cells to progress through the cell cycle. This is in line with Akdemir *et al*.^20^ who found that *Xrp1* expression induces cell cycle arrest in cultured *Drosophila* cells. Since Xrp1 possesses a sequence-specific DNA binding domain (Fig. S5), either one or both of these cellular functions might be directly regulated at the transcriptional level.

**Figure 4:**
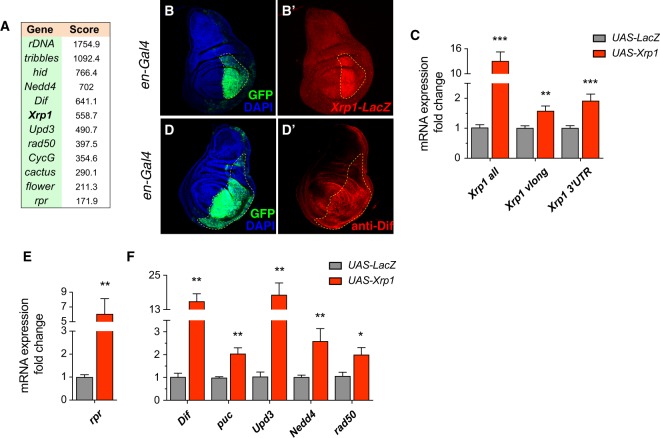
Xrp1 regulates its own expression, the expression of pro-apoptotic genes and of genes previously linked to cell competition. ChIP-seq on Xrp1 OE wing discs reveals targets of Xrp1, including rDNA, *Xrp1* itself, pro-apoptotic genes such as *hid* and *rpr* and several other genes that have been linked to either cell competition or cell proliferation (**A**). Using a transcriptional reporter for Xrp1 and via the overexpression of Xrp1 in the posterior compartment (*en-Gal4*), immunostaining reveals a positive feedback loop by which Xrp1 regulates its own transcription. Xrp1 (**B**-B’). The observation is confirmed by qPCR, Xrp1 is overexpressed in the wing disc 24 hours before analysis. We used primers that recognize all forms of Xrp1 including the overexpressed isoform and confirmed that the Xrp1 overexpression construct is functional (*Xrp1 all*). With primers that detect only the endogenous forms of Xrp1 we observe Xrp1-dependent induction of Xrp1 expression (*Xrp1 vlong*, *Xrp1 3*′*UTR*) (**C**). A representative example showing upregulation of the Dif protein upon overexpression of Xrp1 in the posterior compartment (**D**-D’). Effects on putative Xrp1 target genes are confirmed via qPCRs. The pro-apoptotic gene *rpr* is upregulated in response to Xrp1 OE in wing discs. Xrp1 OE is induced 24 hours before analysis (**E**). Other putative target genes are also upregulated under the same conditions, respectively *Dif*, *puc*, *Upd3*, *Nedd4 and rad50* (**F**). t-test was applied. *P < 0.05; **P < 0.01; ***P < 0.001.

To further explore this notion we set out to identify direct genomic targets of Xrp1 by chromatin immunoprecipitation followed by deep sequencing (ChIP-seq) on wing imaginal discs^29^. In order to do this, we induced *Xrp1* expression in wing discs. The top targets revealed by ChIP-seq comprise a number of genes that are already implicated in cell competition, cell cycle regulation and apoptosis^7,30–32^. Figure 4A shows a list of the most interesting genes that are bound by Xrp1. Among these we identified *Xrp1* itself, suggesting the existence of a potential autoregulatory loop. To test this notion we overexpressed Xrp1 in the posterior compartment of the wing disc and checked the transcriptional behavior of *Xrp1* with the aforementioned *Xrp1-lacZ* reporter. We observed the upregulation of *lacZ* expression in response to Xrp1 overexpression, indicating that Xrp1 can boosts its own expression in a positive autoregulatory loop (Fig. 4B-B’). We confirmed these observations by measuring mRNA levels of *Xrp1* upon forced *Xrp1* expression (Fig. 4C). With a similar strategy we also checked the response of other putative transcriptional targets from our ChIP-seq experiment. We could show that Xrp1 promotes the transcription of *Dif* (Fig. 4D-D’,F), a *Drosophila* NFkB homolog gene that has previously been implicated in the cell competition-dependent induction of apoptosis via the induction of *rpr* transcription^7^. We also tested *puc*, *Upd3*, *Nedd4* and *rad50*: all of these genes were upregulated upon induction of *Xrp1* expression (Fig. 4F). puc, Upd3 and Nedd4 are involved in the JAK/STAT and Hippo signaling pathways, both of which have previously been implicated in cell competition^10,13,14,28,31^. Rad50 is instead required for double strand break repair^33^.

The most prominent sequence motif of Xrp1 derived from ChIP-seq data shows a strong similarity with the b-ZIP binding motif of the human C/EBP protein family. We therefore checked whether Xrp1 shows homology to C/EBP transcription factors, being itself a *bona fide* transcription factor. We found that Xrp1 shares a 40% identity with the human C/EBPs (PSI-BLAST). Phylogenetic reconstruction allowed us to recognize three *Drosophila* C/EBP homologs, one of which is Xrp1 (Fig. S5). Interestingly, human C/EBP-alpha is retained in the nucleolus and binds to ribosomal DNA^34^, a feature that may be evolutionarily conserved since Xrp1 binds rDNA loci with high affinity (Fig. 4A). The encoded rRNA is found in the nucleoli.

**Figure 5:**
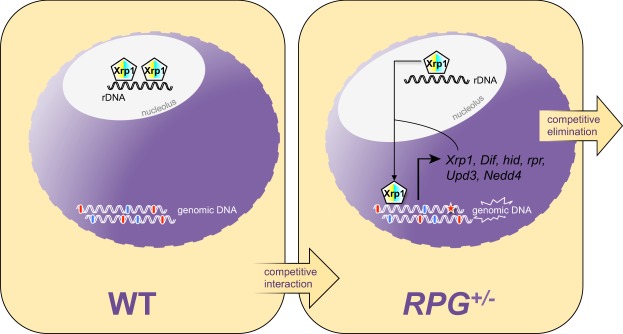
Role of Xrp1 in cell competition-driven elimination of loser cells. Xrp1 localized in the nucleulus sitting on rDNA. In a competitive scenario, Xrp1 acts as a transcription factor in loser cells, driving the expression of *Xrp1* itself, of pro-apoptotic target genes (*hid*, *rpr*), of genes involved in innate immunity (*Dif*), in compensatory proliferation (*Upd3*) and in protein degradation-dependent apoptosis and cell proliferation arrest (*Nedd4*). Xrp1, because of its double involvement in the elimination of loser cells and in DNA repair, putatively acts as a genomic caretaker in a p53-independent fashion.

We therefore propose a working model in which Xrp1, under normal conditions, sits on rDNA in the nucleolus. In the presence of genotoxic stress or of a ribosomal imbalance, as in the context of *Minute* cell competition, Xrp1 acts nuclearly as a C/EBP transcription factor that stimulates its own transcription and the expression of pro-apoptotic target genes (Fig. 5). When intermingled with wild-type cells, cells with only one copy of an *hRPG* are eliminated in a Xrp1-dependent manner. In our experimental system the deletion of one copy of the *RpL19* gene is catalyzed by the Flp/FRT recombination system, which leaves no apparent lesion in the DNA^35^. Therefore, the initial recruitment of Xrp1 into the nucleus may not depend on DNA damage *per se*, but rather on the unbalanced physiology of the cell resulting from the loss of one copy of the *hRPG*. The nucleolus is the site of ribosome biogenesis and a major stress sensor organelle^36^. *RpL19*^+/−^ cells experience a related nucleolar stress, since their nucleoli are enlarged as revealed by anti-fibrillarin staining (Fig. S6). The most likely explanation for this is partially stalled ribosome assembly^37^. Since genotoxic stress triggers *Xrp1* expression (Fig. S6B,C), we speculate that Xrp1 acts as a caretaker of genomic integrity. In support of this hypothesis, the growth of *salvador*^−/−^ mutant tumor clones is suppressed by the concurrent loss of one copy of the *RpL19* gene. However, this suppression fails in the absence of Xrp1 function (Fig. S6), indicating that the presumptive protective function that RPGs haploinsufficiency provides can also operate within tumorous cells. In addition, according to our Monte-Carlo simulation, the likelihood that one *hRPG* locus becomes heterozygous mutant before any other gene gets mutated to homozygosity is very high (Fig. S6). Together with the observation that *hRPGs* are broadly distributed within the genome^38^ (Fig. S6), this further supports the potential role of Xrp1 as a caretaker of genomic integrity. Although further research is required to better elucidate this phenomenon, we nevertheless propose that RPG haploinsufficiency provides a simple, yet effective, mechanism to protect the organism from the emergence of potentially deleterious cells.

## Materials and Methods

### *Drosophila* strains and cultures

Flies were grown on a standard cornmeal medium at 25 °C unless otherwise specified. The *salE-GAL4* (*2*^*nd*^), the *P{EP}dIAP1* and the *en-GAL4*, *UAS-mCD8::GFP*, *tubP-GAL80*^*ts*^ lines were generated in our laboratory. The *UAS-Xrp1*.*ORF*.*3xHA* (*ZH-86Fb*) line was obtained from FlyORF. The *UAS-E2F*, *UAS-DP* (*2*^*nd*^), *Xrp1-LacZ*^*02515*^, *Act5C* > *y* + > *GAL4*, *Df*(*2R*)*M60E*, *P{lacW}RpL19*^*k03704*^, *FRT82B*, *FRT80B*, *tub-GAL80*, *ubi-GFP-nls* (*3*^*rd*^) lines were obtained from the Bloomington *Drosophila* Stock Center. The *UAS-CycEg* (*2*^*nd*^)^39^ and *UAS-cycD*, *UAS-cdk4* (*2*^*nd*^)^40^ were provided by Christian Lehner. The *UAS-mCherry-CAAX* (*2*^*nd*^)^41^ was obtained from Shigeo Hayashi. The *P{PZ}hid*^*05014*^ and the *Dronc*^*O1*^, *FRT80B* stocks were provided by Wei Du^42^. *Xrp1*^*GS18143*^ (#200976) was obtained from the DGRC Kyoto stock center. The *UAS-p35* (*2*^*nd*^)^43^, the *UAS-Rab5* (*2*^*nd*^)^44^ the *UAS-puckered* (*2*^*nd*^)^45^, the *sav*^4^ ^46^ were additionally used. *Act5C* > *GAL4* was obtained by flipping out the *y*+ FRT cassette of *Act5C* > *y* + > *GAL4*. *Bub3-dsRNA-GD9924* (*2*^*nd*^) was obtained from VDRC.

### Cloning and transgenesis

The *RpL19* 3.08 kbp genomic rescue (2R:24967017..24970096 Dmel_r6.08) was amplified from a genomic DNA template. After sequence confirmation it was cloned within the NotI restriction site of the pUAST.attB and inserted into the attP landing site *ZH-attP-86Fb* (3R tester line) and *ZH-attP-68E* (3L tester line)^47^. The *Xrp1* 15.88 kbp BamHI-BglII genomic rescue (3R:18911505..18927381 Dmel_r6.08) was digested from CH321-38O16 of the P[acman] BAC Libraries^48^. After sequence confirmation it was cloned into the pattB vector^49^ and inserted into the *attP* landing site *ZH-attP-68E*. The *Xrp1* mutated genomic rescue was generated by inserting 5 bp (C > GATCCC at 3R:18925226 Dmel_r6.08) at the beginning of the second coding exon in the wild-type genomic fragment, which shifts the frame of all *Xrp1* isoforms. Transgenesis was performed according to standard germ-line transformation procedures.

### Mutagenesis and screen

EMS screens were performed according to standard procedure^50^. *y w hs-FLP; M{3xP3-RFP*.*attP}ZH-36B; FRT82B* starter line was first isogenized for the 3R cell competition screen. Isogenized males were fed with a 25 mM, 1% sucrose solution and crossed to tester virgin females. *RpL19*^+/−^ clones were induced in the resulting progeny. A total of 20,000 F1 larvae were screened for the persistence of *RpL19*^+/−^ GFP positive clones at the end of the third instar larval stage. 182 larvae showed persistence of GFP clones clearly above background noise. 125 of them gave rise to fertile adults and were further rescreened. 12 heritable suppressors were doubly balanced. For the *Xrp1* coding sequence directed mutagenesis *y w;; Xrp1*^*GS18143*^/TM3.Sb males were fed with a 50 mM, 1% sucrose solution and crossed to tester virgin females *y w ey-FLP; Act* > *y* + > *GAL4-w; M{3xP3-RFP*.*attP}ZH-86Fb*. 10,000 F1 genomes were screened and 8 heritable suppressors were retrieved and balanced. A mutation in the *Xrp1* coding region was identified in 5 of them. After the causative mutation was identified the upstream *P{GSV6}Xrp1*^*GS18143*^ was removed using P element transposase and precise excision events were selected (direct sequencing of PCR amplicons) and recombined onto a *FRT82B* chromosome for clonal analysis. *RpL19* knock-out was generated by mobilizing the P element *P{lacW}RpL19*^*k03704*^, imprecise excisions were selected based on the presence of the characteristic *Minute* bristle phenotype and the absence of the *white*^+^ marker. The *RpL19*^*IE-C5*^ 1.09 kbp deletion (2R:24968426..24969517 Dmel_r6.08) was selected and characterized using direct sequencing of PCR amplicons. This specific excision removes *RpL19* coding sequence and leaves neighboring genes unaffected.

*RpL19*^+/−^ loser clones for *in vivo* screen were generated as follows: *y w hs-FLP; M{3xP3-RFP*.*attP}ZH-36B; FRT82B* mutagenized males were crossed to *y w UAS-mCD8::GFP*, *hs-FLP; salE-GAL4*, *Df*(*2R*)*M60E; FRT82B*, *tubP-GAL80*, *M{RpL19 genomic}ZH-86Fb*/ *SM5a-TM6B* tester virgin females.

### Mapping the mutations

We initially mapped cell competition suppressors through meiotic recombinations coupled with DHPLC (Denaturing High-Performance Liquid Chromatography) for PCR amplicon analysis. The interval containing the suppressors *Xrp1*^*08*^ and *Xrp1*^29^ was narrowed down to a 106.5 Kb interval (3R:18872668..18979166 Dmel_r6.08). Sanger sequencing of the coding regions in this interval did not reveal the presence of any mutation. We then performed whole-genome sequencing on *Xrp1*^*08*^, *Xrp1*^20^ and *Xrp1*^29^ with the Illumina’s Genome analyser IIx (Genomics Platform of the University of Geneva). Mutations were identified by visual inspection of the sequences in this interval: *Xrp1*^*08*^ (T > A 3R:18921364 Dmel_r6.08), *Xrp1*^20^(C > T 3R:18920194 Dmel_r6.08), *Xrp1*^29^(G > A 3R:18921450 Dmel_r6.08). Other suppressors were roughly mapped to the second chromosome or to one of the arms of the third chromosome as indicated in the test complementation table. *Minute* mutants were identified on the basis of their characteristic bristle phenotype and developmental delay. *warts* and *salvador* mutants were identified on the basis of their clonal overgrown phenotypes and failure to complement independent loss of function alleles (*warts*^*m72*^ and *sav*^4^). Note that for *sup*^88^ the suppressive mutation is the *Minute* on the second chromosome and not the mutation in the *salvador* gene. *Xrp1* suppressors isolated from the coding sequence directed mutagenesis were identified by direct sequencing of PCR amplicons: *Xrp1*^*02*^ (G > A 3R:18926271 Dmel_r6.08), *Xrp1*^26^ (C > T 3R:18926088 Dmel_r6.08), *Xrp1*^37^ (C > T 3R:18926394 Dmel_r6.08), *Xrp1*^39^ (C > T 3R:18925431 Dmel_r6.08), *Xrp1*^*61*^ (TC > ACA 3R:18925609..18925610 Dmel_r6.08).

### qRT-PCR

qRT-PCR was performed according to standard protocol. RNA was extracted with TRIzol Reagent and genomic DNA was digested with the Ambion DNase kit. RNA was isolated from third instar wing imaginal discs with the exception of the reaction to evaluate the expression of the different splicing variants in WT and *Xrp1*^*08*^ mutant conditions. In this experiment we used the following primers (primer sequences are oriented 5′ to 3′).

Pr_1: GCGTAGCAGAAAAGACAAGTGA; Pr_2: CGACACAAGTTCCCCTTAAAC; Pr_3: TCATTGTTTCTTTCTAACGGTCAA; Pr_4: GGTTGCTGTTGTTTGATTCG; Pr_5: CCTACTGCCACAGTTGAAGAGATAGACG; Pr_6:TTGCTTCTATGTCTTGCAGGTATT; Pr_7:GACCACACCGGAGATTATCAA; Pr_8: GCTGGTACTGGTACTTGTGGTG.

Pr_1 and Pr_2 were used for Xrp1_GCA; Pr_2 and Pr_3 were used for Xrp1_BGA; Pr_1 and Pr_6 were used for Xrp1_E; Pr_3 and Pr_6 were used for Xrp1_C; Pr_5 and Pr_6 were used for Xrp1_BGAF; Pr_7 and Pr_8 were used for Xrp1_all. For measuring Xrp1 target genes in loser cells large clones overexpressing Xrp1 were induced. To achieve this, males of the aforementioned *UAS-Xrp1:HA* line were crossed with female virgins of the *y w hs-FLP*, *Act* > *CD2* > *Gal4*, *UAS-GFP* line. Heat shock was induced for 45′ at 37 °C, 4 days AED to induce recombination in most of the cells of the wing disc. The following primers were used.

Act5c_fw: CGCCTTGAATTTGTTAAATCG; Act5c_rev: ACATGCCAGAGCCGTTGT; Xrp1_156_fw: CAGCTCCTGAATGATGATCG; Xrp1_156_rev: ATGTCTGCATGGGTGCTG; Xrp1_VL_fw: CGGGATGTGAGTGGAGCAAT; Xrp1_VL_rev: GACGTTGCTTCTATGTCTTGCA; puc_fw: GAGAAGCGTGCGAAGGAG; puc_rev: TTGGGATAGTCCTTCTGATTGG; Upd3_fw: CCCAGCCAACGATTTTTATG; Upd3_rev: TGTTACCGCTCCGGCTAC; rpr_fw: CGAAGAGGTCATCTCCCAAG; rpr_rev: GGGGAACAAAAGCAGGAAA; hid_fw: GTGGAGCGAGAACGACAAA; hid_rev: TTGGCCAAGTGAAGCTCTGT; Xrp1_3′UTR_fw: CGTTGAAGAAGTCGAGAAGCA; Xrp1_3′UTR_rev: TAAACACTCCTCGCGCACTA; Xrp1_GCA_fw: GCGTAGCGAAAAGACAAGTGA; Xrp1_GCA_rev: CGACACAAGTTCCCCTTAAAA; Xrp1_D_fw: TTTTGGTCCGCGGATAAATCT; Xrp1_D_rev: ACGTTGCTTCTATGTCTTGCA; Xrp1_B_fw: AAGGAGCAACAAGGATCAAGA; Xrp1_B_rev: CACAAGTTCCCCTTAAACCTCC; Xrp1_F_fw: CAACCACGTAACCACCCATCT; Xrp1_F_rev: GGGATCTCGAGGATACGCCTG; Xrp1_ACEG_fw: TCATCGCGGAACAATAACAGTG; Xrp1_ACEG_rev: GCAATAGGTTGGGTGGTTCC; Dif_fw: GTGGAGCTGAAACTAGTGAGACC; Dif_rev: GGCGATTGTGTTTGGTTAGG; Nedd4_fw: GACCCTGGTGAATCTGCCTA; Nedd4_rev: CCGGATAAAGGCGTGGTAG.

### ChIP-seq preparation and analysis

Wing imaginal discs expressing HA-tagged *Xrp1* (FlyORF-F000655)^48^ were mass isolated and sorted, chromatin was immunoprecipitated and DNA libraries were prepared according to standard protocol^29^. Rabbit anti-HA ChIP grade antibody (ab9110, Abcam) was used. Libraries were sequenced on the Illumina HiSeq. 2500 v4 (Functional Genomics Center of the University of Zurich). Bowtie 2 (version 2.0.0-beta6)^51^ was used to align the sequencing reads using default parameters. The dm3 *Drosophila* genome annotation was used as reference. The program findPeaks.pl with default parameters was used to identify enriched regions compared to the untreated control sample. The program MotifsGenome.pl (with the option size = 75) was used to identify predominant motifs. The sequence logo was generated with the PWM-Tools web interface (http://ccg.vital-it.ch/pwmtools/) from the SIB using HOMER’s position frequency matrix output file.

### Clone induction, immunostaining and imaging

*RpL19*^+/−^ loser clones for dissections: males of the appropriated genotype were crossed to the “3R” or “3L” tester virgin females. “3R” tester virgin females: *y w hs-FLP; Act5C* > *GAL4-w*, *UAS-mCherry-CAAX*, *Df*(*2*R)*M60E; FRT82B*, *ubi-GFP-nls*, *tubP-GAL80*, *M{RpL19 genomic}ZH-86Fb*/*SM5a-TM6B*. “3L” tester virgin females: *y w*, *hs-FLP; Act5C* > *GAL4-w*, *UAS-mCherry-CAAX*, *Df*(*2R*)*M60E; ubi-GFP*.*D*, *tubP-GAL80*, *M{RpL19 genomic}ZH-68E*, *FRT80B*/ *SM5a-TM6B*. Parents were allowed to lay eggs for 24 hours and *RpL19*^+/−^ loser clones were heat-shock induced for 30 minutes at 37 °C, 24–48 hours AED. Progeny were screened at the end of the third instar larval stage when larvae stop feeding and move away from the food. No water was added nor was heat-shock applied to force the remaining larvae out of the food as it is routinely done.

*RpL14*^+/−^ loser clones: *y w hs-FLP;;RpL14*^−^ *SalE* >*RpL14* > *Gal4 UAS-mCD8::GFP*, *Xrp1*^*61*−^/*TM6b* virgin females were crossed with respectively *y w*, *Xrp1*^*61*−/−^ and *UAS-RpL14* males. Clones were heat shock-induced for 15′ at 37 °C, 48 hours AED.

Immunostainings on wing discs were performed according to standard protocol. The following antibodies were used: rabbit anti-Cleaved-Caspase-3 (Asp175, Cell Signaling), mouse anti-β-Galactosidase (Z3781, Promega), mouse anti-Fibrillarin (38F3; Santa Cruz). The rabbit anti-Dif antibody was obtained from Ylva Engström, the monoclonal mouse anti-Hid antibody was obtained from Hermann Steller. The following secondary antibodies were used: goat anti-mouse Alexa Fluor 647, goat anti-rabbit Alexa Fluor 647 and goat anti-rat Alexa Fluor 647 (Molecular Probes).

Wing discs were imaged using a Leica LSM710 confocal microscope.

### Quantification and statistics

Statistical analyses were performed in Graphpad Prism 7 or Microsoft Excel. Depending on the distribution of data, t-test or Mann-Whitney test were used, unless differently indicated. Regarding *RpL19*^+/−^ loser clones for dissections and clone size quantification, we undertook a stringent comparative analysis based on the ratios between the areas of loser (mCherry) and winner (GFP^2+^) clones. Areas were quantified with FIJI. We applied standardized statistical tests (Mann-Whitney test). In addition, we reasoned that a genuine suppressor of *RPG* mutant cell elimination should not only increase the mean size of *RPG* mutant clones but also restore a normal distribution of *RPG* mutant clones (in this case statistical analysis was performed by using the D’Agostino & Pearson normality test). For *RpL14*^+/−^ loser clones, GFP area was measured with FIJI and Mann-Whitney test was applied.

Signal intensity calculation for Xrp1 targets was performed in FIJI with the mean gray intensity measurement tool. Statistical significance was calculated with a paired-ratio t-test.

### Generation of imaginal wing discs with *RpL19*^+/−^ and *RpL19*^+/+^compartments

*y w hs-FLP; Act5C* > *GAL4-w, UAS-mCherry-CAAX, Df*(*2*R)*M60E*/ *RpL19*^*IE-C5*^*; FRT82B, ubi-GFP-nls, tubP-GAL80, M{RpL19 genomic}ZH-86Fb*/ *FRT82B* larvae were heat-shocked 15 min at 37 °C during L1. Wing discs were dissected at the end of the third instar larval stage, fixed, stained and imaged.

### Identification of Xrp1 homologs

In a heuristic approach, two iterations of PSI-BLAST^52^ were performed using the bZIP domain of Css as a query. The COBALT constraint-based multiple protein alignment tool provided on the BLAST interface^53^ was used to align all *Drosophila* Xrp1 protein sequences with the human C/EBPs family members identified with the PSI-Blast search. In a non-heuristic approach, BZip containing proteins from human and *D*. *melanogaster* were searched, aligned and trimmed according to the bZIP_2 motif from Pfam (PF07716) using probabilistic hmmer profiles^54^ (hmmer.org). The resulting alignment was visualized with CLC Main Workbench and then used for phylogenetic reconstruction using the PhyML algorithm^55^ with LG substitution models^56^, SPR topological rearrangements^57^ and 100 bootstrap replicates. Phylogenetic tree was then mid-point rooted and displayed with the iTOL online tool^58^.

### *Drosophila RPGs* map/gene density

Gene coordinates for each chromosome arm were retrieved using the cytosearch tool of Flybase. Gene positions were considered as the middle point between the start and the end of each gene. Gene density was calculated for 40 kbp bins and the final map was visualized using the radar chart type of Excel. The percentage of intragenic sequences was calculated as the complement of the total size of the genome minus the sum of the intergenic sequences downloaded from Flybase (Genome, FTP, r6.1).

### Monte-carlo simulation for *RPGs* as caretakers of genomic integrity

The computational model was realized with Phyton (detailed code is provided with the supplementary material). The Monte-carlo simulation was designed to determine the probability that a certain number of different genes is disturbed (both alleles are mutated) when a certain number of random mutations occur. It is assumed that each allele has the same probability to be hit by a mutation and that each mutation hits an allele.

## Electronic supplementary material

Supplementary Dataset 1
