# Supplementary material for: Xrp1 is a transcription factor required for cell competition-driven elimination of loser cells
Source: Sci Rep. 2018 Dec 7;8:17712. doi: 10.1038/s41598-018-36277-4 (PMC6286310; doi:10.1038/s41598-018-36277-4)
Supplement: Supplementary file 1 — Supplementary Dataset 1 [file 41598_2018_36277_MOESM1_ESM.pdf]

**Supplementary file**

# Xrp1 is a transcription factor required for cell competition-driven elimination of loser cells


**Authors**

Ludovic Baillon[1], Federico Germani[1*], Claudia Rockel[1], Jochen Hilchenbach[1] and Konrad Basler[1*]

[1]Institute of Molecular Life Sciences, University of Zurich, Switzerland.

*corresponding authors: federico.germani@uzh.ch, kb@imls.uzh.ch

## Supplementary figures

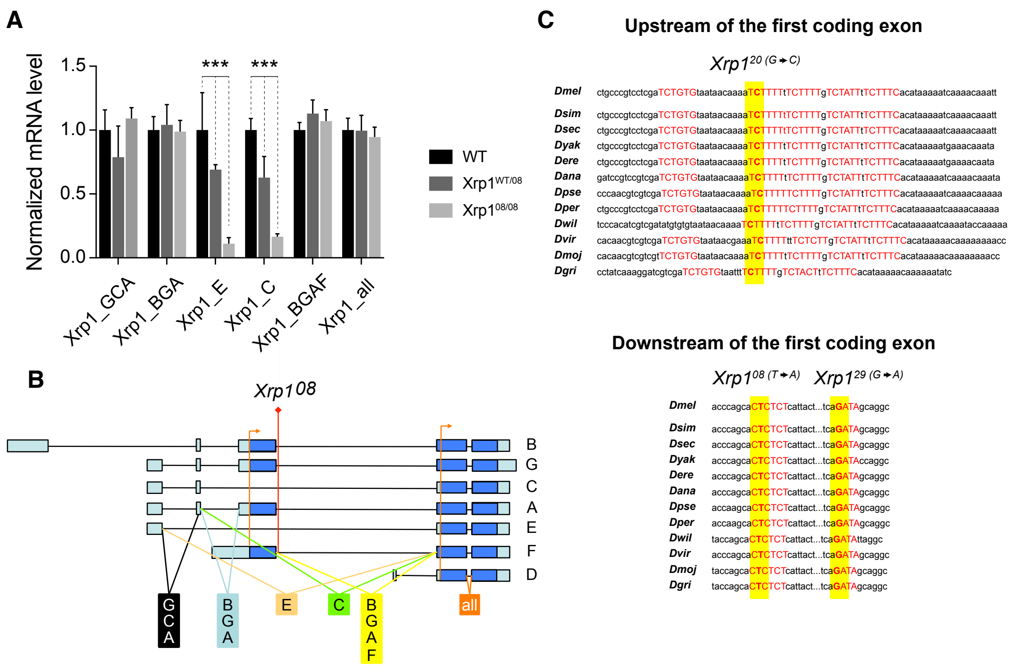


**Figure S1. EMS-retrieved intronic mutations are important for the splicing of *Xrp1*.** Representative qPCR reveals that *Xrp1*$^{08}$ leads to a constitutive downregulation of the transcripts E and C, but not of the transcripts GCA, BGA, BGAF and all (**A**). Schematic representation of the different *Xrp1* splicing isoforms and of the combinations of primers used for the qPCR (**B**). Alignment of *Drosophila Xrp1* sequences. The mutations retrieved from the EMS are indicated with the nucleotide substitutions. Conservation of these nucleotides is extended to intronic motifs, which are capitalized and depicted in red. *Xrp1*$^{20}$ disrupts the repetition of the conserved hexanucleotide pyrimidine motif TCTDTB. *Xrp1*$^{08}$ disrupts the conserved putative intronic splice enhancer (ISE) CTCTCT and *Xrp1*$^{29}$ disrupts a conserved GATA motif (**C**).

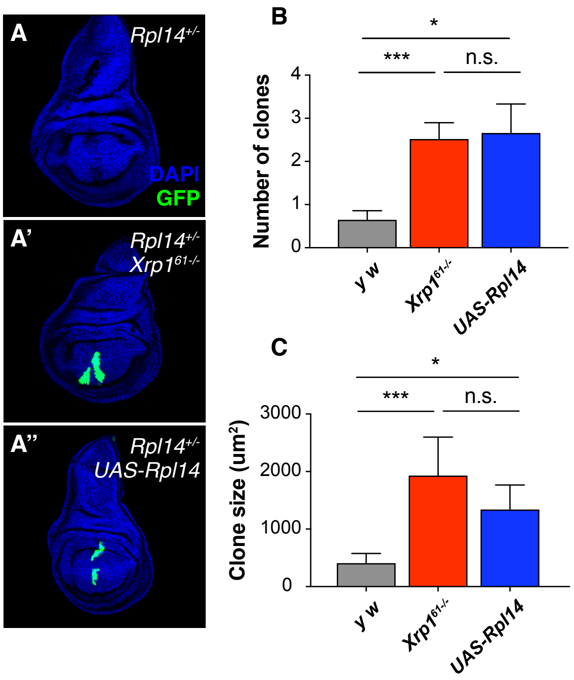


**Figure S2. Xrp1 is required for the elimination of *RpL14*$^{+/-}$ loser cells.** *RpL14*$^{+/-}$ loser cells are eliminated from the wing pouch (SalE driver) via cell competition **(A)**. *Xrp1*$^{61-/-}$ rescues the elimination of *RpL14*$^{+/-}$ loser cells **(A')** similarly to a positive control rescue obtained via the overexpression of *RpL14* **(A'')**. *Xrp1*$^{61-/-}$ (in red) rescues both the number **(B)** and the size **(C)** of *RpL14*$^{+/-}$ GFP$^{+}$ clones. ***P<0.001, **P<0.01, n.s.=not significant. Kruskal-Wallis test. Bars represent SEM. n=19,19,8 (B and C).

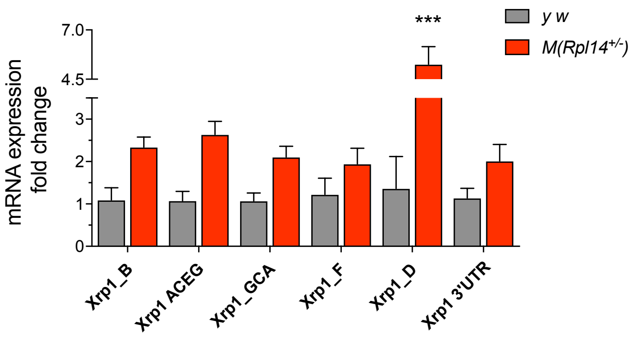


**Figure S3. Different Xrp1 isoforms are upregulated in *RpL14*$^{+/-}$ wing discs.**
qPCR analysis shows that different isoforms of *Xrp1* are upregulated in *RpL14*$^{+/-}$ wing discs. t-test was applied. ***P<0.001.

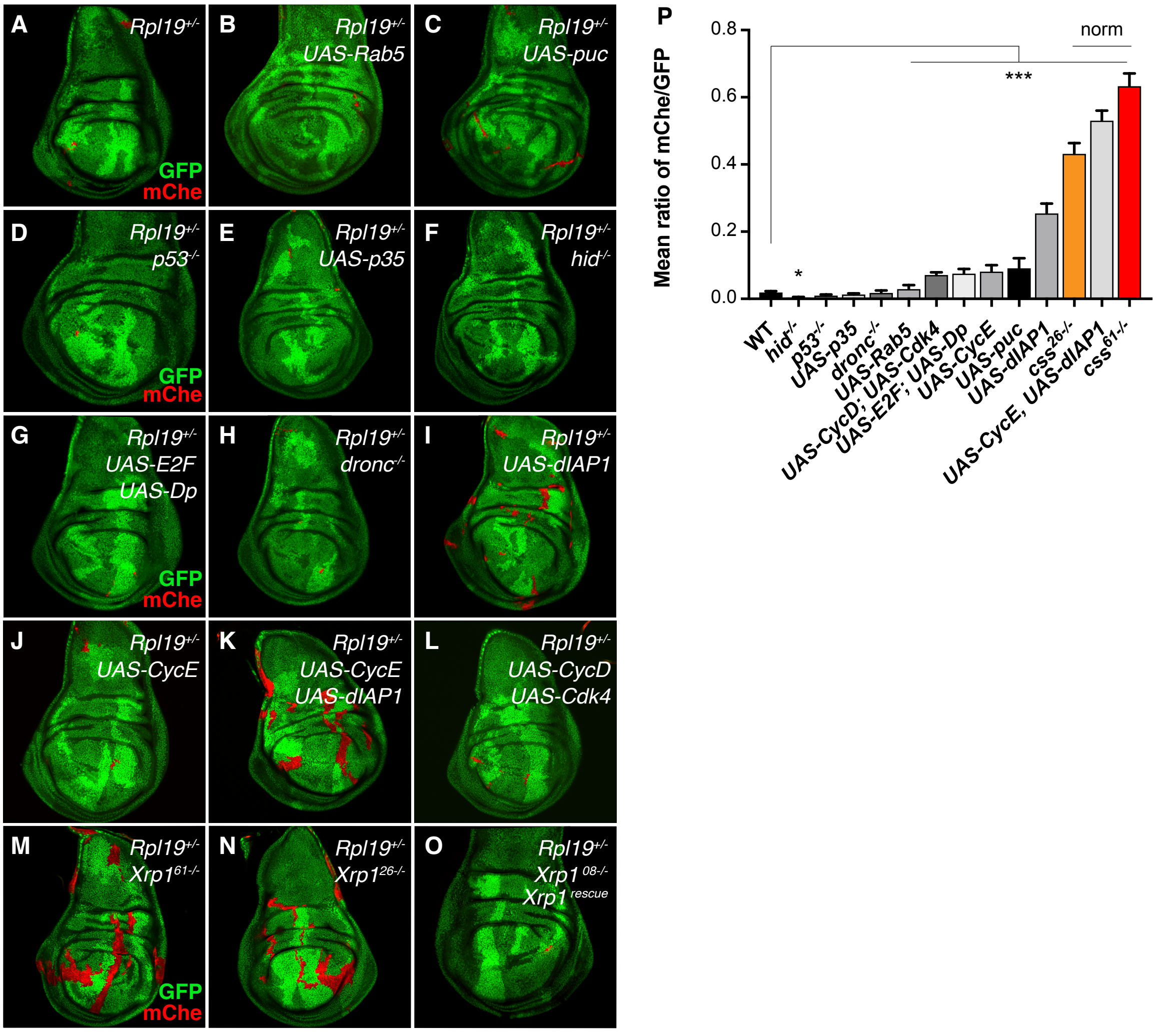


**Figure S4. *Xrp1* is a very strong suppressor of cell competition.** Representative discs displaying the suppressive potential of various genetic alterations. Loser clones are labeled with mCherry. Cell competition-driven elimination of $RpL19^{+/-}$ loser cells **(A)** is not rescued with the overexpression of either *Rab5* **(B)** or *puc* **(C)**, with the removal of *p53* **(D)**, with the overexpression of *p35* **(E)**, with the removal of *hid* **(F)**, the concomitant expression of *E2F* and *Dp* **(G)** or the depletion of *dronc* **(H)**. Partial rescue is obtained with the overexpression of *dIAP1* **(I)**, no rescue is obtained with *CycE* overexpression **(J)**. However, overexpression of *CycE* and *dIAP1* together potentiates the rescue efficacy of *dIAP1* overexpression alone **(K)**. No rescue is obtained with double overexpression of *CycD* and *Cdk4* **(L)**. Strong suppression of the elimination is obtained with various alleles of *Xrp1*, in the representative examples with $Xrp1^{61}$ **(M)** and $Xrp1^{26}$ **(N)**. When a copy of *Xrp1* is reintroduced, loser cells are eliminated **(O)**. This indicates that Xrp1 is the strongest suppressor of cell competition and that the elimination of *minute* cells requires multiple inputs, particularly the induction of apoptosis and cell cycle arrest. **(A-O)** Quantification of the mean mChe/GFP ratio. Genotypes are ranked according to their suppressive potential. ***P<0.001, *P<0.05. Mann-Whitney test. Bars represent SEM. n=66,50,58,23,44,24,57,61,26,27,27,50,81,46 (in order of suppressive potential) (A to O). "norm" indicates the normality of the distribution as tested with the D'Agostino & Pearson normality test (P>0.05 indicates normality). $Xrp1^{61}$ (p=0.135), *UAS-CycE;*

*UAS-dIAP1* (p=0.125) and *Xrp1*$^{26}$ (p=0.052). For all the other genotypes the probability density functions are extremely right skewed with (P<0.0066) **(P)**.

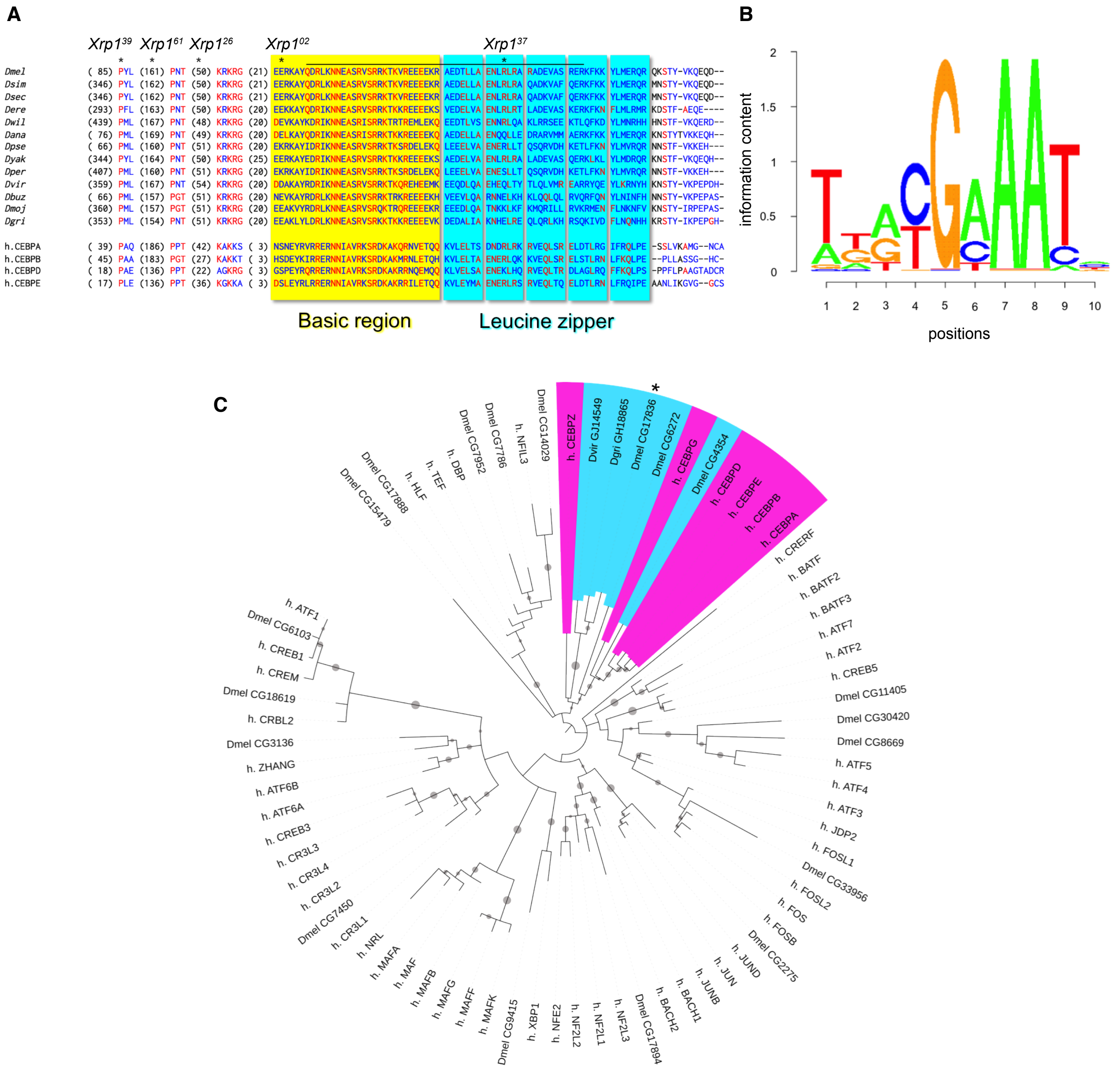


**Figure S5. Xrp1 is homolog of human C/EBPs.** Alignment of *Drosophila* Xrp1 protein sequences with the human C/EBPs. Sequences with a minimum of 3 contiguous similar amino acids (AAs) are depicted; red color indicates identity, blue indicates similarity and black means difference; the numbers in brackets indicate the length of AAs that do not align. Asterisks indicate the positions of the EMS alleles mapped to the *Xrp1* open reading frame: $Xrp1^{39}$ (P86S), $Xrp1^{61}$ (D144>FS), $Xrp1^{26}$ (Q269!), $Xrp1^{02}$ (E330K), $Xrp1^{37}$ (R371!). The line above the Xrp1 sequence shares a 40% identity with the human C/EBPs (PSI-BLAST). Highlighted in yellow is the basic region (DNA binding), in blue is the leucine zipper (hetero/homo-dimerization) of the basic region-leucine zipper domain (b-ZIP) **(A)**. Sequence logo of the most prominent motif bound by Xrp1 in the ChIP-seq experiment (present in 38% of the peaks, P≤1e-1009). This motif relates to the b-ZIP binding motif of the C/EBPs

protein family **(B)**. Phylogenetic reconstruction using PhyML and visualized with iTOL. Branches with a bootstrap value superior to 80% are marked with a grey round sign. Humans C/EBPs are highlighted in magenta and *Drosophila* C/EBPs homologues are highlighted in blue. Xrp1 position is marked with an asterisk **(C)**.

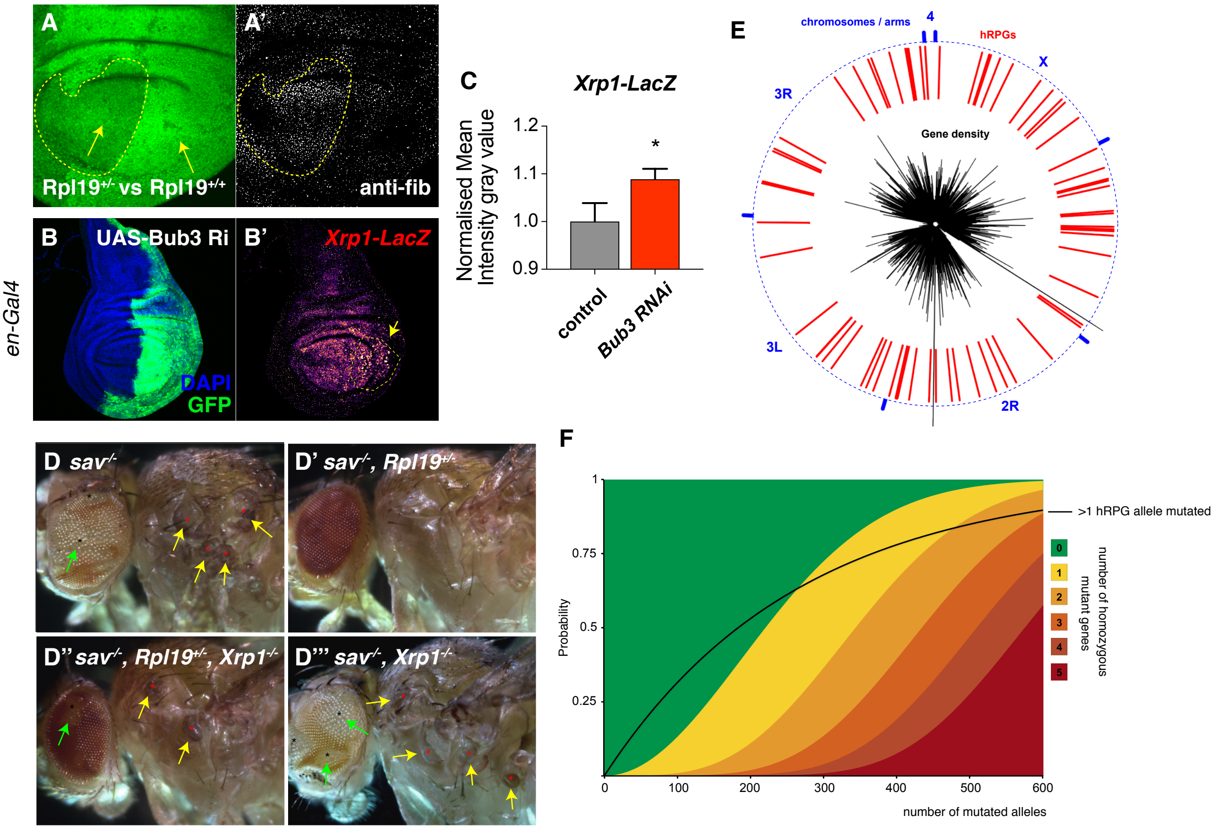


**Figure S6. Xrp1 is a caretaker of genomic instability.** *Rpl19*$^{+/-}$ compartment shows enlarged nucleoli as observed with an anti-Fibrillarin staining (**A-A'**). Genomic instability obtained with the downregulation of Bub3 in the posterior compartment of the wing disc induces *Xrp1* expression, as revealed with a LacZ reporter (**B-B'**), quantified with the normalized mean intensity grey value (**C**). Xrp1 exerts a tumor suppressive function. *salvador*$^{-/-}$ mutant flies develop tumors (**D**) and their generation can be rescued when animals are additionally heterozygous mutant for *RpL19* (**D'**). Additional abrogation of Xrp1 function suppresses the anti-tumorigenic effect of *RpL19* mutations (**D''**). *sav*$^{-/-}$, *Xrp1*$^{-/-}$ flies also develop tumors (**D'''**). Wide distribution of hRPGs in the *Drosophila* genome (**E**). Monte-carlo simulation shows that the probability that both alleles of a random gene are mutated is lower than the probability of having 1 *hRPG* allele mutated when the number of mutated alleles is not extremely elevated (**F**).

## SM file 1 – Phyton code for Monte-Carlo simulation

@author: tinria

```
"""
import numpy.random as rd
import numpy as np

simno=1000#1000 #number of simulations, the higher this number, the more precise the probability
maxk=600#600 #maximum number of mutations to be analyzed
n=35016#35016 #number of different alleles
max_pairs=5 #the probability is given to get 0 pairs, 1 pairs, 2 pairs ... max_pairs pairs
probs_tot=''#in this string all results are saved; per entry of the list: first
#number: number of mutations simulated, second number: probability that no
#pair is hit, third number: probability that one pair is hit, etc
print 'k, from 0 pairs until '+str(max_pairs)+' pairs'
for k in range(maxk):
    pairs=np.zeros(simno)
    pairs2=np.zeros(simno)
    for i in range(simno):
        genes=np.zeros(k)
        alleles=np.zeros(k)
        genes=rd.randint(0,n//2,k)
        alleles=rd.randint(0,2,k)
        freq_gene_hit=np.bincount(genes)
        genes_hit_at_least_twice=np.where(freq_gene_hit>=2)[0]
        for gene in genes_hit_at_least_twice:
            pos=np.where(genes==gene)[0]
            num=5
            change=0
            for l in range(len(pos)):
                if alleles[pos[l]]!=num:
                    change+=1
                    num=alleles[pos[l]]
            if change>=2:
                pairs[i]+=1

    probs=np.zeros(max_pairs+1)
    print str(k),
    for i in range(max_pairs+1):
        probs[i]=len(np.where(pairs==i)[0])/simno
        print str(probs[i]),
    probs_tot+=str(k)+' mutations:'+str(probs[:]).strip('[').strip(']')+'\n'
    print
print probs_tot
```
